# Genotypic and phenotypic variation in transmission traits of a complex life cycle parasite

**DOI:** 10.1002/ece3.621

**Published:** 2013-06-04

**Authors:** Katja-Riikka Louhi, Anssi Karvonen, Christian Rellstab, Jukka Jokela

**Affiliations:** 1 Department of Biological and Environmental Science, University of JyväskyläP.O. Box 35, FI-40014, Jyväskylä, Finland; 2 Swiss Federal Research Institute WSLZürcherstrasse 111, CH-8903, Birmensdorf, Switzerland; 3 Eawag, Swiss Federal Institute of Aquatic Science and TechnologyP.O. Box 611, CH-8600, Dübendorf, Switzerland; 4 ETH Zürich, Institute of Integrative BiologyCH-8092, Zürich, Switzerland

**Keywords:** Bet hedging, host condition, host–parasite interaction, phenotypic plasticity, Trematoda

## Abstract

Characterizing genetic variation in parasite transmission traits and its contribution to parasite vigor is essential for understanding the evolution of parasite life-history traits. We measured genetic variation in output, activity, survival, and infection success of clonal transmission stages (cercaria larvae) of a complex life cycle parasite (*Diplostomum pseudospathaceum*). We further tested if variation in host nutritional stage had an effect on these traits by keeping hosts on limited or ad libitum diet. The traits we measured were highly variable among parasite genotypes indicating significant genetic variation in these life-history traits. Traits were also phenotypically variable, for example, there was significant variation in the measured traits over time within each genotype. However, host nutritional stage had no effect on the parasite traits suggesting that a short-term reduction in host resources was not limiting the cercarial output or performance. Overall, these results suggest significant interclonal and phenotypic variation in parasite transmission traits that are not affected by host nutritional status.

## Introduction

Studies on life-history traits let us expect that many of the phenotypic differences observed among individuals have a genetic basis (Roff 1992; Stearns 1992). There are no reasons to assume that parasite life-history traits would be an exception of this pattern (Poulin 1996), although evolution of parasite life-history traits is less studied using the concepts of life-history theory. This may be surprising because understanding the processes that maintain genetic variation in transmission-related traits is fundamental for our understanding of how parasite transmission evolves. Transmission, on the other hand, is a key factor underlying evolution of parasite virulence (defined here as harm to the host) as well as epidemiological dynamics of infection and impact of parasites on host populations.

Parasites that reproduce clonally at some stage of their life cycle provide interesting opportunities to test how much of the phenotypic variation has a genetic basis. Clonal stages allow testing of how much of the variation in phenotypic traits among the same genotype results from environmental effects (Falconer and Mackay 1996). The comparison of genetically identical individuals has classically been used in twin studies, in experiments with clonal plants and animals, and more recently also with clonal parasite stages (see e.g., Anderson et al. 2010; Koehler et al. 2011). Variation among genotypes reflects the opportunity for natural selection to operate on life-history trait expression, whereas a high variability within a genotype reflects phenotypic plasticity or classical bet-hedging strategies that are beneficial in stochastic environments (Fenton and Hudson 2002; Kussell and Leibler 2005; Donaldson-Matasci et al. 2008; Reece et al. 2009).

One potential mechanism maintaining variation in life-history traits of parasites is genotype-by-environment interactions (G × E). The speciality of complex (indirect) life cycle parasites is that in some cases genetic variation among parasite genotypes includes the effects of the interaction with unique host genotypes. Such an intimate interaction is particularly common in parasites with complex life cycles where propagules produced by sexual reproduction (egg or miracidia larva) infect host genotypes that also originate from sexual events. These unique parasite–host genotype combinations are not replicated and both the genetic and phenotypic effects of the host genotype become part of the parasite genotype's extended phenotype. Nevertheless, it is valuable to evaluate how much of the variation in trait expression among parasite genotypes is due to genetic effects, even if the contribution of host genotype cannot be separated from the direct effects of the parasite genotype. The magnitude of genetic variance among parasite genotypes allows to evaluate how much of the fitness differences among parasite genotypes are caused by external environment. In evolutionary ecology such genetic variation that includes both heritable and nonheritable variation is coined in measures of “broad-sense” genetic variability (see e.g., Falconer and Mackay 1996).

The special features that the intimate interaction of parasites with their host and their external environment creates motivate studies of their evolutionary ecology. Developmental conditions (e.g., individual quality, immune status, and number of conspecific parasites) within the hosts tend to vary substantially, and often unpredictably, among different host individuals (Poulin 2007; Reece et al. 2009). Therefore, genetically identical parasites are not necessarily phenotypically similar as they have to deal with different host environments (Poulin 1996). Conditions for parasite development have important implications for initial infection success, within-host infection dynamics, and transmission success to the next host. On one hand, it is generally assumed that hosts in good condition are hostile environments for parasites as they are effective in resisting infections. On the other hand, hosts in bad condition might provide fewer resources for parasite growth and reproduction (Ebert 2000; Bedhomme et al. 2004; Ebert et al. 2004), and the resulting shorter duration of infection (due to eventual death of the host) could lead to reduced parasite fitness. The effect of host condition on host susceptibility (Krist et al. 2004) and the effect of environmental stress on parasite virulence (Jokela et al. 1999, 2005; Pulkkinen and Ebert 2004) have been studied intensively, but studies concerning the effect of host condition on parasite traits have received less attention (but see Logan et al. 2005; Tschirren et al. 2007). For example, in snail–trematode interactions host individuals supplied with low-quality food release fewer larval stages compared with hosts provided with high-quality diets (Keas and Esch 1997; Sandland and Minchella 2003; Seppälä et al. 2008). This suggests that host exploitation by parasites is dependent on host condition, which again could determine the quality of larval stages (Seppälä et al. 2007, 2008).

In this study we focus on the variation in life-history traits of trematodes, a group of parasites with a complex life cycle typically including three transmission steps, an endothermic final host, a mollusc first intermediate host, and an invertebrate or vertebrate second intermediate host. Trematodes show distinctive behavioral (reviewed by Combes et al. 1994; Haas 1994), physiological, and morphological traits (Koehler et al. 2011), which are potentially important during transmission between the hosts. Transmission-related traits of larval stages (e.g., cercarial output, activity, survival, and infection success) are good candidates for characters that determine transmission success and subsequent parasite fitness as they affect the likelihood that the parasite reaches its final host. However, maximizing all traits simultaneously is practically impossible due to physiological and ecological constraints (Partridge and Harvey 1988). For example, natural selection could favor parasite genotypes that produce high numbers of clonally produced larval transmission stages in the first intermediate host, as this could increase the probability that the genotype will successfully encounter the second intermediate host. On the contrary, a high investment in asexual reproduction could be constrained by low quality of the larvae or increased host mortality (Davies et al. 2001) and decrease parasite fitness. For free-swimming parasite stages such as trematode cercariae, active movement is an important determinant of transmission success as the parasites must localize and enter the next host. However, increased energy (glycogen) consumption through movement shortens their life span (Ginetsinskaya 1988) as these larval stages do not feed. The longevity of the parasite larvae is important as longer survival increases the time that the parasite genotype can use to localize the subsequent host. Moreover, a successful parasite genotype should be infective, but infectivity may trade-off with other traits as shown in a snail–schistosome system, where cercarial production in the snail host was negatively correlated with infectivity to a mouse definitive host (Davies et al. 2001).

In this study, we measured genotype-level variation in transmission-related traits of *Diplostomum* parasites and studied experimentally how host (freshwater snail, *Lymnaea stagnalis*) nutritional status affects these traits. We measured traits from parasites originating from field-collected snails that were manipulated for their nutritional status by dividing them into two food treatments (“ad libitum food” and “no food”) for 2 weeks. The food manipulation was applied to examine how differences in host condition affect parasite traits and to identify possible trade-offs between the traits as allocation theory predicts that trade-offs should be most evident when resources are limiting (e.g., Bazzaz et al. 1987).

## Materials and Methods

### Study system

*Diplostomum* (Trematoda) parasites are macroparasites with a complex life cycle that involves freshwater snail (first intermediate host), fish (second intermediate host), and fish-eating bird hosts (final host). Parasites mate in the intestine of fish-eating birds and produce eggs that are shed into water with bird feces. Hatched, free-swimming miracidia infect the snail, where the parasite produces transmission stages asexually. This results in thousands of free-swimming cercariae larvae that leave the snail and infect the eye lenses of the second intermediate fish host (a wide variety of freshwater fish species can be infected, Rellstab et al. 2011). Intensive *Diplostomum* infections cause parasitic cataracts in several species of wild and farmed fish (Karvonen et al. 2004b; Karvonen and Seppälä 2008; Seppälä et al. 2010). The final bird host is infected by predation of infected fish.

### Experimental snails

*Lymnaea stagnalis* is a common freshwater snail occupying shallow littoral zones of stagnant lakes and ponds with lush vegetation. *L. stagnalis* serves as the first intermediate host for *Diplostomum pseudospathaceum* in Finland (Louhi et al. 2010). Snails (*n* = 670) were collected from lake Vuojärvi (62°25′N, 25°56′E) in central Finland between 14th and 16th of July 2009, and brought to laboratory. Cercarial shedding was observed under light microscope and 75 snails that released cercariae of *D. pseudospathaceum* were identified. Fifteen larvae per infected snail were genotyped at three polymorphic microsatellite loci Diplo06, Diplo09, and Diplo23 (Reusch et al. 2004) to separate snails that were infected either with one (*n* = 55) or multiple parasite genotypes. Details of the genotyping methods are described in Louhi et al. (2010). During the genotyping the snails were kept individually in 1 L of water at <5°C and fed *ad libitum* with lettuce for 1 week.

Ten days prior to the experiment only the snails infected with one parasite genotype were placed individually in 1.5 L floating plastic containers. The containers were perforated with small holes that allowed flow through of water and randomly distributed in eight 470 L tanks with closed circulating lake water running through sand filters. Water temperature was 15°C and snails were kept under a natural light–dark cycle (16L:8D). The snails were fed lettuce ad libitum to maximize their condition. After this 10-day acclimatization period 32 snails (mean shell length ±SE = 53 ± 1 mm) were randomly chosen and divided into two treatment groups, “*ad libitum*” and “no food,” leaving 16 snails for each treatment group. Snail size (*t*_30_ = −0.853, *P* = 0.401) was not significantly different between the treatment groups. The “*ad libitum*” group continued on an *ad libitum* diet, whereas the “no food” group did not receive any food. Afterward, the containers were checked daily for mortality and they were reshuffled among the tanks four times during the experiment to exclude tank effects. Three hours before monitoring of the cercarial traits (see below), snails were placed individually in jars with 0.25 L of lake water (22°C) and allowed to release cercariae.

It is important to note that as we used field-infected snails we could not control for the age of infection or have replicates of parasite genotypes in different host genetic backgrounds. We decided to use this approach because we were not able to infect several snail individuals with the same parasite genotype without interfering with the natural life-history trait expression of the parasites. In nature the asexual production of clonal parasite stages takes place only within snails, and larval stages infecting snails are sexually produced in birds. Thus, the effects of the host genetic background and host phenotype on cercarial traits, whatever those might be, remain as integral part of the “broad sense” genetic variation that we measured among the parasite genotypes. It is analog to “maternal effects” affecting the phenotypic expression of offspring traits. Therefore, throughout this study, parasite genotype is defined in a broad sense, including the extended genotype and phenotype of the host. In other words, “parasite genotype” includes the parasite genotype itself, the phenotypic effect of the snail host, and the G × G interaction between host and the parasite genotype. The advantage of our approach is that the broad-sense genetic variation we measure mimics the natural situation. We aimed to minimize the variation in snail condition by feeding all the snails *ad libitum* for 10 days before the experiment (see above) and used manipulation of the host to evaluate the magnitude of host phenotypic effects on parasite life-history trait expression.

### Cercarial traits

Four cercarial traits (cercarial output, swimming activity, survival, and infection success in the next host) were determined for each parasite genotype. This was done before the food treatment started (referred to as week 0 below), after 1 week of food treatment (week 1), and after 2 weeks of food treatment (week 2). We chose the starvation period of the snails based on our previous studies that have shown that 2 weeks of starvation can have a significant effect on parasite traits (Seppälä et al. 2008). However, in this study, contrary to Seppälä et al. (2008), snail survival (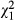 = 1.032, *P* = 0.310) was not significantly different between the treatment groups in the end of the experiment.

### Cercarial output

The total number of cercariae produced by each parasite genotype (snail) in 3 h was estimated by taking five 1 mL subsamples from each jar and counting the larvae under a microscope. The counting of the produced cercariae from the snails was done by randomizing the order of snails each time. Similar randomization was done also for the determination of the other traits; activity, survival, and infection success (see below).

### Cercarial activity

Three cercariae (maximum 3 h old) of each parasite genotype were placed individually in 250 μL of water (22°C) on a 96-well flat-bottom plate. Genotypes were assigned randomly to each row. Cercariae maintain themselves in the water column by making a short swimming burst which is followed by a resting phase. This behavior repeats at intervals of few seconds. The number of swimming bursts (observed under a microscope) executed in 2 min was counted for each cercaria and used as a measure of cercarial activity for each genotype.

### Cercarial survival

Sixteen parasite cercariae (maximum 3 h old) per genotype were placed individually in 250 μL of lake water (22°C) on a 96-well flat-bottom plate. The clones were assigned randomly to each row. Survival of the cercariae was observed under a microscope after 24 h (Seppälä et al. 2008). Cercariae that did not move were considered dead. The percentage of surviving cercariae was then calculated for each parasite genotype.

### Cercarial infection success

Cercarial infection success was determined using rainbow trout (*Oncorhynchus mykiss*) fry that were obtained from a fish farm using a ground water supply. This ensured that the fish were free of *D. pseudospathaceum* infections before the experiment. However, the fish were subsequently held in tanks with lake water where they became infected to a low degree during the progression of the experiment (weeks 0–2). The level of this background infection was monitored by dissecting a sample (*n* = 14–18) of control fish after each infection trial. It is important to note that each parasite genotype used in the trials faced similar mean background infection in the fish, which is why a previous infection was unlikely to influence the result. However, it may have had an effect on the level of infection among weeks through host immunization and we discuss this possibility in more detail below.

In each experiment, ten randomly chosen fish (mean length in experiment 1 ± SE = 107 mm ±1, in experiment 2 ± SE = 109 mm ±1, and in experiment 3 ± SE = 115 mm ±1) were exposed to cercariae from each parasite genotype. Fish length was not significantly different between the treatment groups (two-way ANOVA: *F*_1,929_ = 0.086, *P* = 0. 769), but increased over time (*F*_1,929_ = 41.99, *P* < 0.001). The fish were placed individually in containers with 0.5 L of water (17°C) and 100 cercariae for 30 min. After the exposure, all fish from one parasite genotype were placed in a plastic 40 × 40 × 40 cm mesh cage. The cages were randomly distributed in four 1500-l tanks supplied with lake water (17°C) for 48 h to allow parasite establishment in the eye lenses. Afterward, all fish were killed with an overdose of 0.01% MS 222 (Sigma Chemical Co., St. Louis, MO) and the number of parasites was determined by dissecting the eye lenses. The total number of parasites in the right and left eye lense was used as an estimate of infection success for the given parasite genotype.

### Ethical note

The parasite dose used in the experimental infections was based on our earlier experiments (e.g., Karvonen et al. 2003) and the resulting number of parasites in the eye lenses corresponded to that observed in the wild (e.g., Rellstab et al. 2011). The experiment used a total of 1157 fish and the mortality of the fish was low (0.3%). The experiment was carried out with permission from Finnish Regional State Administrative Agency (license number ESLH-2008-05,938/Ym-23) and it conformed to the animal care legislation of Finland.

### Broad-sense heritability

Broad-sense heritability (*H*^2^) is defined as *V*_G_/*V*_P_ (where *V*_G_ is the genotypic variance and *V*_P_ is the phenotypic variance; see e.g., Falconer and Mackay 1996). In clonal parasites the elimination of *V*_G_ can be achieved experimentally as genetically identical larvae can be obtained from a snail infected with one parasite genotype. We used components of variance to estimate *H*^2^, according to the model *V*_G_**/**(*V*_G_ + *V*_E_), where *V*_G_ is the among-genotype variance component and *V*_E_ is the residual phenotypic variance component. The broad-sense heritabilities were calculated only for cercarial activity and infection success as cercarial output data did not include true clonal replicates and because the response variable was binary in cercarial survival data. We tested whether genetic differences among genotypes were significant by calculating the among-genotype variance component with a general linear model.

### Genetic correlations between the traits

Pearson's correlations (*r*) between genotype trait means were used as an approximation of genetic correlation (Via 1991; Vorburger 2005). Transformations were used for cercarial output (ln(x)) and cercarial activity (ln(x + 2)) data to achieve normality.

### Statistical analysis

Results of week 0 were analyzed separately to test for possible differences between the treatment groups before the food treatments started and to determine to what extent there is genotype-specific variation in the traits. Cercarial output, activity, and infection success were analyzed using nested linear-mixed models (LMMs). For cercarial survival a generalized linear-mixed model (GLMM) with binomial response and logit link was used. Results of weeks 1 and 2 were analyzed together, using week as a factor, to test for significant differences in the traits between the treatments and weeks. Also these analyses were done with LMMs and a GLMM, respectively. When needed, response variables were ln-transformed to achieve equal variance and normality. Week and treatment were used as fixed factors and parasite genotype (nested within treatment) as a random factor in the analyses. In the analysis of infection success, fish length was used as an additional covariate. Infection success was analyzed both with and without one outlier parasite genotype (G28). One snail (carrying parasite genotype G41) died after week 1 and therefore this genotype was excluded from the analysis of weeks 1 and 2. Statistical tests were performed with IBM Statistics 20 statistical package (Armonk, NY) using 0.05 as the statistically significant level.

## Results

### Cercarial output

**Figure 1:**
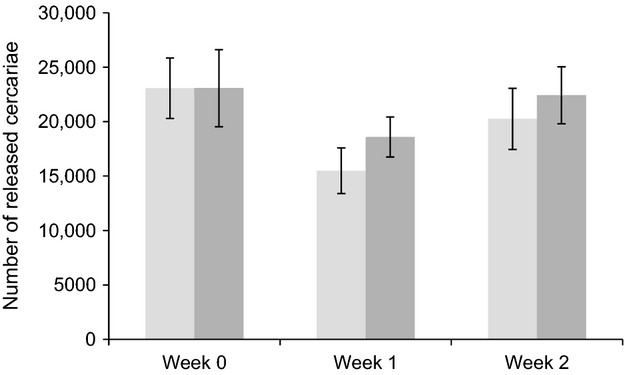
Mean cercarial output ±SE of parasites from normally fed snails (light bars) and starved snails (dark bars). Week 0, all snails received food; week 1, food manipulation had been applied for 1 week; week 2, food manipulation had been applied for 2 weeks.

**Figure 2:**
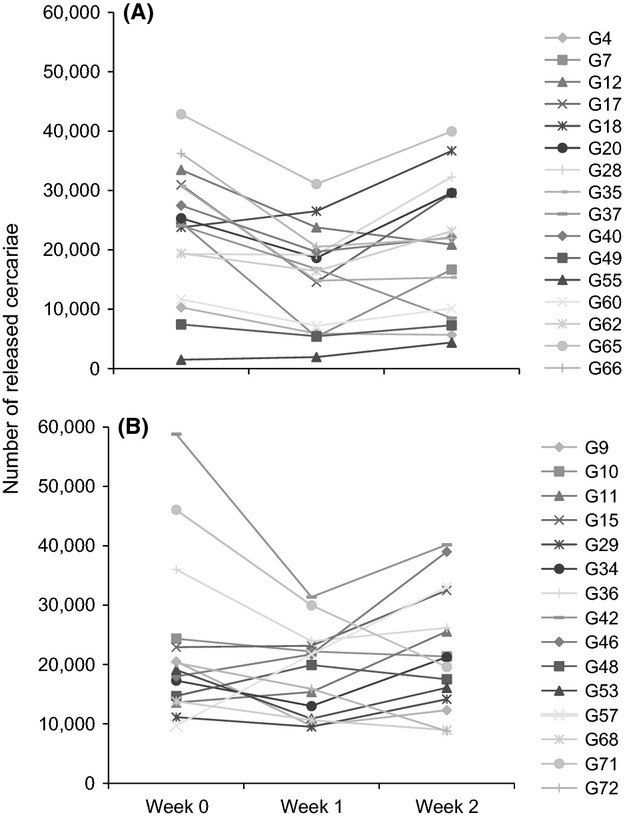
Numbers of cercariae produced in 3 h. *Diplostomum pseudospathaceum* genotypes from normally fed snails (A) and food-deprived snails (B). Food manipulation started after week 0. Week 0, all snails received food; week 1, food manipulation had been applied for 1 week; week 2, food manipulation had been applied for 2 weeks.

Cercarial output did not differ between the treatment groups before the food treatment started (Table 1, Fig. 1), but the differences among the parasite genotypes were pronounced (Fig. 2). Host starvation during the following 2 weeks of the experiment had no effect on cercarial output (Table 2), whereas parasite genotype, week, and their interaction each had statistically significant effects (Table 2; Fig. 2). Parasite genotype had the largest effect size (partial *η*^2^, Table 2) followed by the effect of week-by-genotype interaction, emphasizing among-genotype differences. Indeed, the difference between the worst and the most productive parasite genotype was 29-fold. Reaction norms of the genotypes were crossing over the weeks in the experiment (Fig. 2) illustrating temporal variation in cercarial output. On average the cercarial output was lowest on week 1 increasing to pretreatment levels during week 2.

**Table 1:** Results of the three linear-mixed models (LMMs) (cercarial output, activity, and infection success) and one generalized linear-mixed model (GLMM) (cercarial survival) analyzing trait differences among parasite genotypes before the food treatments were applied

Source	df/df_error_	MS	*F*	*P*	Part *η*^2^
Cercarial output					
Treatment	1/30	0.122	0.041	0.841	0.001
Genotype (treatment)	30/128	2.995	77.255	<**0.001**	0.948
Error	128	0.039			
Cercarial activity					
Treatment	1/30	0.139	0.903	0.349	0.029
Genotype (treatment)	30/64	0.154	2.382	**0.002**	0.528
Error	64	0.065			
Cercarial survival					
Treatment	1/28	–	0.335	0.568	–
Genotype (treatment)	–	0.636^1^	2.439	**0.015**	–
Cercarial infection success					
Treatment	1/30.0	14,091	5.156	**0.031**^2^	0.147
Genotype (treatment)	30/284	2738	9.905	**<0.001**	0.511
Fish length	1/284	11,586	41.904	**<0.001**	0.129
Error	30.0	2733			

Parasite genotype is treated as a random factor nested under food treatment. Partial Eta Squared (Part *η*^2^) indicates effect sizes. One parasite genotype (G28) had approximately nine times lower infection success compared with the other parasite genotypes. Therefore, the analysis of infection success was run with and without this genotype and both results are given. Significant *P*-values (*P* < 0.05) are marked with bold font.

1 Parameter estimate from GLMM. Estimate for week-by-genotype (treatment) effect is redundant and not reported.

2 *P* = 0.055 if outlier genotype 28 is removed from the analysis.

**Table 2:** Results of the three linear-mixed models (LMMs) (cercarial output, activity, and infection success) and one generalized linear-mixed model (GLMM) (cercarial survival) analyzing life-history trait differences among parasite genotypes after 1 and 2 weeks of food treatment

Source	df/df_error_	MS	*F*	*P*	Part *η*^2^
Cercarial output					
Week	1/29	3.635	9.435	**0.005**	0.245
Treatment	1/29	5.241	1.556	0.222	0.051
Genotype (treatment)	29/29	3.368	8.741	**<0.001**	0.897
Week × treatment	1/29	0.265	0.687	0.414	0.023
Week × genotype (treatment)	29/248	0.385	8.146	**<0.001**	0.488
Error	248	0.047			
Cercarial activity					
Week	1/29	0.022	0.131	0.720	0.004
Treatment	1/29	0.062	0.192	0.665	0.007
Genotype (treatment)	29/29	0.324	1.935	**0.040**	0.659
Week × treatment	1/29	0.007	0.044	0.835	0.002
Week × genotype (treatment)	29/124	0.168	2.214	**0.001**	0.341
Error	124	0.076			
Cercarial survival					
Week	1/947	–	0.846	0.367	
Treatment	1/27	–	1.703	0.203	
Genotype (treatment)	–	0.471^1^	2.636	**0.008**	
Week × treatment	1/947	–	4.716	**0.030**	
Error	58	0.131			
Cercarial infection success					
Week	1/30.8	19.586	67.064	**<0.001**	0.686
Treatment	1/29.0	17.890	3.806	0.061^2^	0.116
Genotype (treatment)	29/29.0	4.705	15.398	**<0.001**	0.939
Week × treatment	1/29.0	<0.001	0.001	0.980	<0.001
Week × genotype (treatment)	29/545	0.306	2.615	**<0.001**	0.122
Fish length	1/545	8.829	75.507	**<0.001**	0.122
Error	545	0.117			

Parasite genotype is treated as a random factor nested under food treatment. Partial Eta Squared (Part *η*^2^) indicates effect sizes. One parasite genotype (G28) had approximately 10 times lower infection success compared with the other parasite genotypes. Therefore, the analysis of infection success was run with and without this genotype and both results are given. Significant *P*-values (*P* < 0.05) are marked with bold font.

1 Parameter estimate.

2 *P* = 0.041 when outlier genotype 28 is removed.

### Cercarial activity

**Figure 3:**
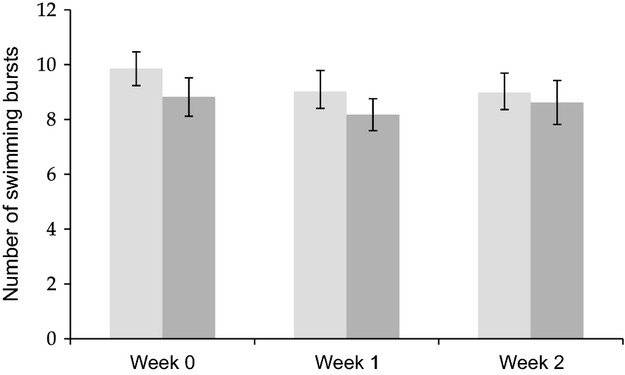
Mean cercarial activity ±SE. Parasites from normally fed snails (light bars) and starved snails (dark bars). Week 0, all snails received food; week 1, food manipulation had been applied for 1 week; week 2, food manipulation had been applied for 2 weeks.

**Figure 4:**
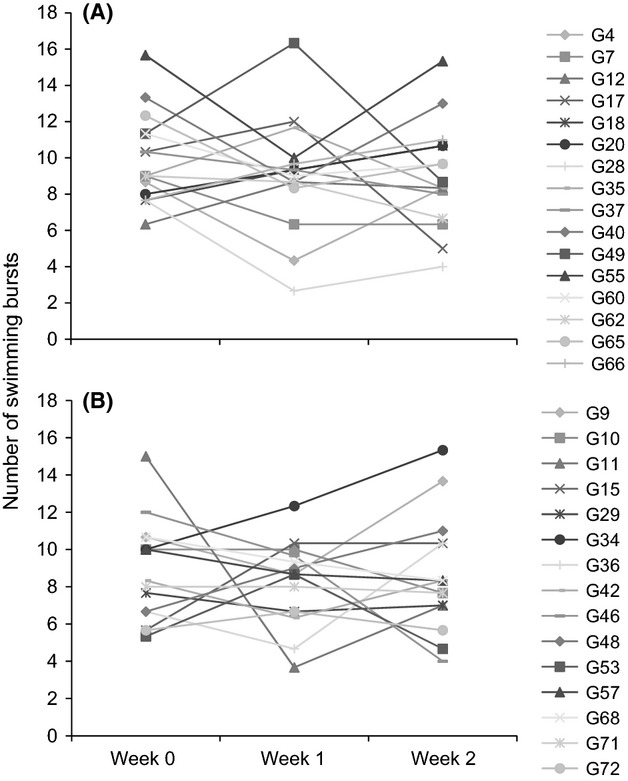
Number of swimming bursts performed in 2 min. *Diplostomum pseudospathaceum* genotypes from normally fed snails (A) and food-deprived snails (B). Food manipulation started after week 0. Week 0, all snails received food; week 1, food manipulation had been applied for 1 week; week 2, food manipulation had been applied for 2 weeks.

Patterns in cercarial activity were along the lines of cercarial output. Activity of cercariae did not differ between the host treatment groups before the food manipulation started (Table 1, Fig. 3) or thereafter (Table 2). The main source of variation for cercarial activity came from parasite genotype, followed by the interaction between parasite genotype and week (Table 2, Fig. 4). There was almost a fourfold difference in the number of swimming bursts between the most and least active genotype. The average activity of cercariae did not differ considerably over time or between the treatments (Fig. 3).

### Cercarial survival

**Figure 5:**
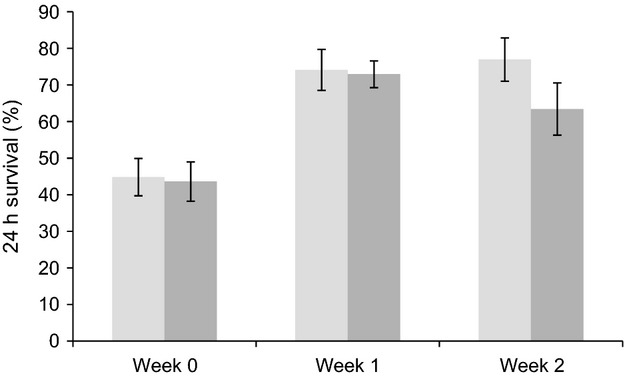
Mean cercarial survival after 24 h ±SE. Parasites from normally fed snails (light bars) and starved snails (dark bars). Week 0, all snails received food; week 1, food manipulation had been applied for 1 week; week 2, food manipulation had been applied for 2 weeks.

**Figure 6:**
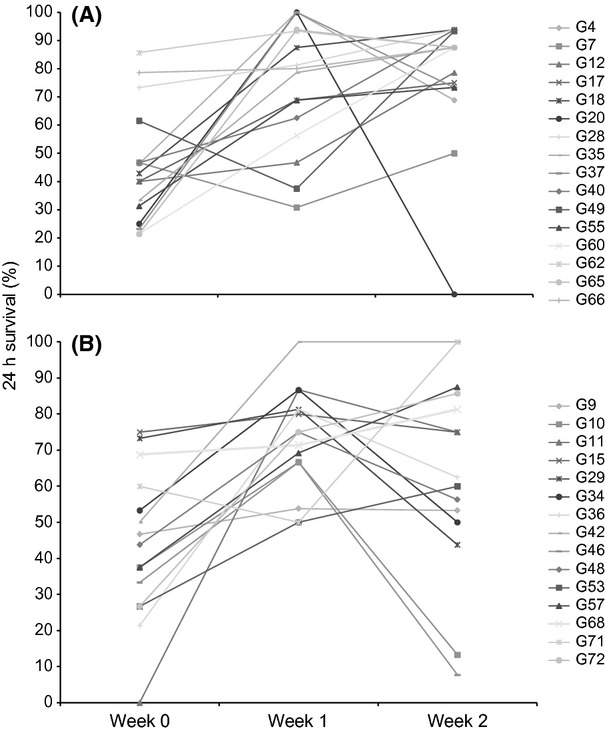
Percentage of alive cercariae after 24 h. *Diplostomum pseudospathaceum* genotypes from normally fed snails (A) and food-deprived snails (B). Food manipulation started after week 0. Week 0, all snails received food; week 1, food manipulation had been applied for 1 week; week 2, food manipulation had been applied for 2 weeks.

Parasite genotype had a significant effect on cercarial survival both before and after the beginning of food manipulation (Tables 2). There were up to 90-fold differences in cercarial survival among the genotypes. As for cercarial output and activity, survival did not differ between the treatment groups before the food manipulation started (Table 1, Fig. 5) or thereafter (Table 2). However, the week-by-treatment interaction had a significant effect on cercarial survival (Table 2), as the cercariae released from starved snails on average had a lower survival after 2 weeks of host starvation (Figs. 5, 6). Overall, the mean survival of cercariae was lowest on week 0.

### Cercarial infection success

**Figure 7:**
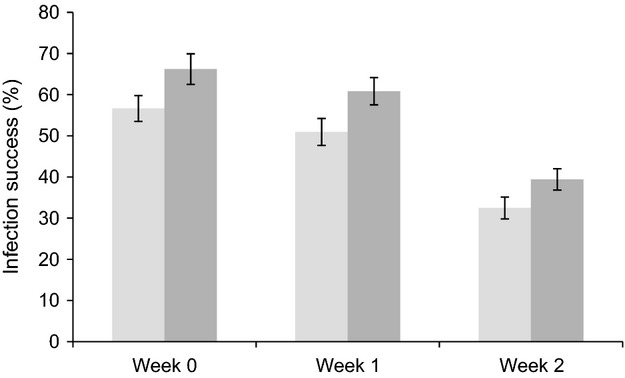
Mean infection success ±SE. Parasites from normally fed snails (light bars) and starved snails (dark bars). Week 0, all snails received food; week 1, food manipulation had been applied for 1 week; week 2, food manipulation had been applied for 2 weeks.

Unexpectedly, we observed a significant difference in the mean infection success between the treatment groups on week 0, already before the food treatment started (*P* = 0.031; Table 1; Fig. 7). The difference was due to a single outlier parasite genotype (G28) (Table 1), which had a very low infection success throughout the experiment. As for all the other traits we measured, also the cercarial infection success was largely dependent on the parasite genotype. The genotype effect on cercarial infection success was, in fact, even larger than the genotype effect on other traits we measured (Partial *η*^2^, Table 2) and the genotypes exhibited up to threefold differences in their infection success. Also the effects of week and genotype-by-week interaction were statistically significant, but weaker than the effect of genotypes (Table 2). Throughout the analyses, fish length was a significant covariate.

**Figure 8:**
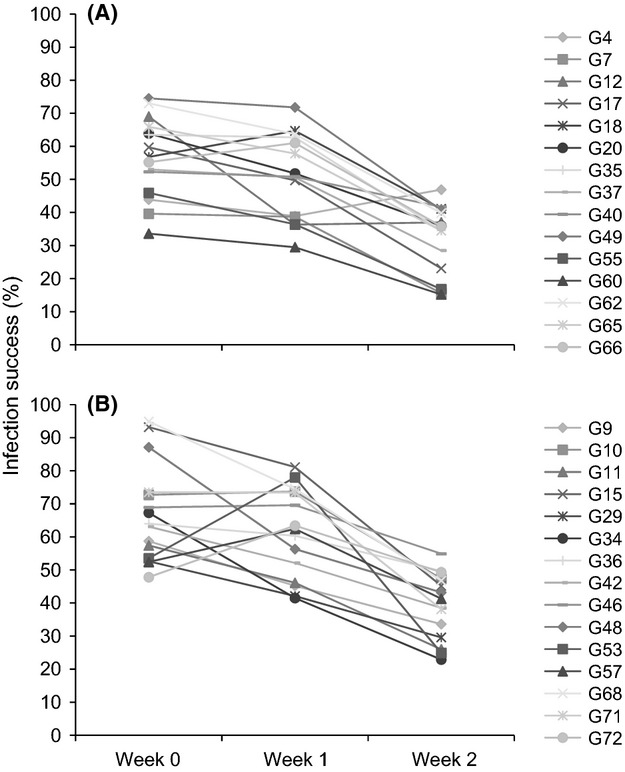
Percentage of cercariae successfully establishing in the fish eye lenses. *Diplostomum pseudospathaceum* genotypes from normally fed snails (A) and food-deprived snails (B). Food manipulation started after week 0. Week 0, all snails received food; week 1, food manipulation had been applied for 1 week; week 2, food manipulation had been applied for 2 weeks.

Although we had unwanted background infections in the fish when they entered the experiment, we believe that this had no effect on our main results. The tanks we held the fish before the experiment were supplied with lake water from the same inlet pipe. In other words, the low exposure level was similar for all experimental fish and thus the treatment groups were comparable. For example, before the experimental exposures the fish carried, on average, 1.2 ± 0.4 (mean ± SE for week 0), 2.0 ± 0.4 (mean ± SE for week 1), and 1.6 ± 0.3 (mean ± SE for week 2) parasites per fish, which is a significantly lower number (*t*-test, *P* < 0.001 in all tests) than in the experimentally infected fish that had, for example, on average 32.5 ± 2.7 (mean ± SE for week 2 in the “*ad libitum*” group) and 39.4 ± 2.6 (mean ± SE for week 2 in the “no food” group) parasites per fish. The result that might be explained by the background infection of the fish is the lower infection success of cercariae in week 2 than on week 0 (see Fig. 7). It is possible that the previous infection induced immune responses in the fish, therefore one should expect the cercarial infection success to decrease from week 0 to week 2 (see Fig. 8).

### Genetic correlation between the traits

A negative correlation was found between cercarial activity and output (*r* = −0.451, *n* = 32, *P* = 0.010) before the food treatments started. After 1 week of treatment infection success was positively correlated with activity (*r* = 0.759, *n* = 16, *P* = 0.001) in the “*ad libitum*” group, but not in the “no food” group (*r* = 0.268, *n* = 16, *P* = 0.316). After 2 weeks of treatment none of the traits showed significant correlations.

### Heritability

Broad-sense heritability estimates for cercarial activity and infection success were relatively high and also quite stable over time in both treatment groups except for the high *H*^2^ (0.67) for cercarial activity found in the “no food” group in week 2 (Table 3).

**Table 3:** Broad-sense heritabilities (*H*^2^) of cercarial activity and infection success

Trait	Treatment	Week	*G*	*n*	*H*^2^	*P*^1^
Cercarial activity	*ad libitum*	0	32	3	0.31	<0.001
	*ad libitum*	1	15	3	0.40	0.005
	No food	1	15	3	0.33	0.015
	*ad libitum*	2	15	3	0.31	0.020
	No food	2	15	3	0.67	<0.001
Infection success	*ad libitum*	0	31	10	0.34	<0.001
	*ad libitum*	1	15	10	0.29	<0.001
	No food	1	15	10	0.27	<0.001
	*ad libitum*	2	15	10	0.38	<0.001
	No food	2	15	10	0.35	<0.001

On week 0 all snails received food, on week 1 food manipulation had been applied for 1 week, and on week 2 food manipulation had been applied for 2 weeks. Number of parasite genotypes used for the estimation of *H*^2^ (*G*) and number of replicates per parasite genotype (*n*).

1 Among-genotype variance component from a general linear model that tests whether genetic differences exist among genotypes.

## Discussion

Transmission-related traits are important determinants of parasite fitness. In addition to genetic differences among parasite genotypes, environmental factors such as the condition of the previous host may affect infection success of the parasite in the next host. In this study we examined genotype-specific transmission characteristics of clonal parasite stages (*D. pseudospathaceum*, Trematoda) and tested if food deprivation of the snail intermediate host affected these traits.

We found considerable variation in all the measured transmission traits among parasite genotypes that originated from naturally infected snails. Depending on the trait, 51–95% of the variance was attributable to parasite genotype in the broad sense that we use it in this study. In addition to parasite genotype, experiment week and genotype-by-week interaction were the most important effects in the statistical tests. These results are in line with those from a marine trematode (*Maritrema novaezealandensis*) that show significant differences among cercarial clones in morphology, photoreactive behavior, and survivorship (Koehler et al. 2011), as well as with those from malaria parasites (*Plasmodium* sp.) for which substantial intraspecific life-history variation is commonly found (e.g., Mackinnon and Read 1999; Eisen 2000; Eisen and Schall 2000). Moreover, a recent study by Rieger et al. (2013) found large differences among four different clonal lines of *D. pseudospathaceum* in traits such as overall hatching success, time of hatching, and infection rates in snails and three-spined sticklebacks. Taken together, these results suggest that genotype-level variation in transmission traits is common in many parasite species.

Although we observed relatively high (ranging between 0.27 and 0.67) and stable *H*^2^ estimates of cercarial activity and infection success, the genotypes showed different mean trait values on different weeks, indicating that genotypes switched ranks during the experiment in both treatment groups. As *H*^2^ estimates include the genetic and phenotypic variation introduced by the host individuals, the estimates are literally clonal repeatabilities including variation that usually would be estimated under maternal and epistatic effects (see below). Therefore, our data give evidence for high clonal repeatability within the clones measured at a given time point, but the phenotypic effects of the snail host may also contribute to the large week-to-week phenotypic variation within the parasite genotypes. Thus, variation in transmission traits within a genotype may be adaptive or nonadaptive for the parasite, but requires further study.

The variance of a trait may be itself subjected to selection and parasites may exhibit different levels of variability in life-history traits as a result of selection in hosts or habitats that differ in predictability (Poulin 2007). *D. pseudospathaceum* parasites infect several different freshwater fish species (Rellstab et al. 2011) and have highly motile birds as final hosts which ensure high levels of parasite gene flow among snail populations (Louhi et al. 2010). In such a situation intraclonal variability could assure that at least a small proportion of the phenotypes always match to the available hosts (Cosseau et al. 2010). This idea is known as bet-hedging strategy (reviewed by Hopper 1999) and could be useful in variable environments, where it could maximize the likelihood that some clones will survive and transmit successfully (Fenton and Hudson 2002; Kussell and Leibler 2005; Donaldson-Matasci et al. 2008).

Another possibility is that the observed variation is nonadaptive for the parasite and mainly reflects temporal host–parasite dynamics in the snail. Although we minimized phenotypic snail effects by keeping all snails on an *ad libitum* diet for considerable time period before the experiment and by using snails infected with only one parasite genotype, it was not possible to control for all snail effects. Thus, variation among the parasite genotypes also includes the genetic and phenotypic effects of the snail host (Eisen and Schall 2000). For example, the age of infection in the snail host could affect parasite traits, if the amount of resources that are available for the parasite changes when the infection spreads in host tissue (Seppälä et al. 2008). However, if the amount of resources declined in some of the experimental snails, we would have expected to see a gradual decline in the parasite trait values in such snails and no significant weekly variation in trait values as observed here. Therefore, it is more likely that temporal dynamics related to cercarial production in the snail could have affected the parasite traits. Such dynamics could be caused by differences in the infection phase of the snails as trematode infections typically alternate between cercarial output and production of new daughter sporocysts within the snails (Ward et al. 1988; Kechemir and Théron 1989; Touassem and Théron 1989). For example, cercarial performance could be determined by the level of synchrony in cercarial release and host energy level (see Karvonen et al. 2004a). Such temporal variation stresses the challenge of measuring parasite fitness–related traits in practice as a measurement taken in one time point might or might not correlate with the lifetime performance of that particular clone. If such “maternal effects” of the snail explain the differences then parasite genotypes should show substantial phenotypic plasticity in response to these temporal dynamics in the snail. Unfortunately, it was not possible to separate effect of the genetic and “snail maternal effects” as it would require an experimental design where several snail individuals are infected with the same parasite genotype. However, the advantage of our approach is that the broad-sense genetic variation that we measured gives a more realistic estimate of the variation present in natural parasite populations.

The importance of selection on traits that contribute to transmission success is difficult to estimate without detailed understanding how particular traits interact (Agnew and Koella 1999). For example, we found a trade-off between cercarial output and activity (at the beginning of the experiment) and a positive correlation between activity and infection success (“*ad libitum*” group, week 1). The trade-off between activity and cercarial output indicates that parasite quality may be constrained when the parasite invests resources into reproduction. The positive correlation observed between activity and infectivity, on the other hand, suggests that those parasite genotypes that are actively swimming have also higher infection success in the next host. However, the instability or inconsistency of the genetic correlations indicates that the positive and negative genetic correlations may vary over time due to environmental effects (see Gutteling et al. 2007), such as the temporal dynamics in the snail. Alternatively, it may suggest that trade-offs do not constrain evolution of these traits. Interestingly, no correlations or trade-offs were observed in the “no-food” group. This is somewhat contradictory as trade-offs should become more likely under unfavorable conditions (e.g., Bazzaz et al. 1987). In this case the scarcity of genetic correlations in weeks 1 and 2 might depend on the smaller sample size (*n* = 15–16 vs. 32 on week 0) or on weak treatment effects as discussed below.

The manipulation of food availability for the snail (*L. stagnalis*) host of *D. pseudospathaceum* explained only a small fraction of the variation observed in parasite life-history traits. The treatment groups did not differ in mortality of the snails, which might indicate that the 2-week food manipulation was not enough to cause severe starvation in the snails collected from Lake Vuojärvi. However, in our previous experiment that used snails from another lake, L. Huumojärvi, similar food deprivation increased the mortality of the snails and decreased the number of released cercariae from the “starved” snails (Seppälä et al. 2008). The difference between these results may be explained by the larger snail size in Vuojärvi compared with Huumojärvi (shell length 53 mm vs. 44 mm), when snails might have more resources to resist short periods of food stress. In addition, in this study, the snails were kept in lower ambient temperature (15°C in this study vs. 20°C in Seppälä et al. 2008), which might have reduced snail metabolism and led to weaker treatment effects.

In summary, these data reveal that transmission-related life-history traits of *D. pseudospathaceum* are highly variable within a naturally infected snail population. This is in line with the high levels of neutral genetic diversity reported for this trematode species (Louhi et al. 2010). The variation in parasite traits was not affected by a 2-week starvation treatment of the snail hosts. Instead, we found significant differences among the genotypes in all four transmission traits examined as well as significant temporal variation in the traits within each genotype. This may be indicative of an adaptive bet-hedging strategy, where temporal within-genotype variation increases the likelihood of some parasite clones surviving and reaching the next host. Alternatively, such variation may result from phenotypic plasticity in response to temporal dynamics within the snail hosts.

## Data Accessibility

Life-history trait data is deposited in the Dryad Repository doi: 10.5061/dryad.dq7ks
